# Impact of kinase activating and inactivating patient mutations on binary PKA interactions

**DOI:** 10.3389/fphar.2015.00170

**Published:** 2015-08-18

**Authors:** Ruth Röck, Johanna E. Mayrhofer, Verena Bachmann, Eduard Stefan

**Affiliations:** Institute of Biochemistry and Center for Molecular Biosciences, University of InnsbruckInnsbruck, Austria

**Keywords:** molecular interactions, patient mutations, cAMP-dependent protein kinase A, protein-fragment complementation assay, GPCR, Carney complex, Acrodysostosis

## Abstract

The second messenger molecule cAMP links extracellular signals to intracellular responses. The main cellular cAMP effector is the compartmentalized protein kinase A (PKA). Upon receptor initiated cAMP-mobilization, PKA regulatory subunits (R) bind cAMP thereby triggering dissociation and activation of bound PKA catalytic subunits (PKAc). Mutations in PKAc or RIa subunits manipulate PKA dynamics and activities which contribute to specific disease patterns. Mutations activating cAMP/PKA signaling contribute to carcinogenesis or hormone excess, while inactivating mutations cause hormone deficiency or resistance. Here we extended the application spectrum of a Protein-fragment Complementation Assay based on the *Renilla* Luciferase to determine binary protein:protein interactions (PPIs) of the PKA network. We compared time- and dose-dependent influences of cAMP-elevation on mutually exclusive PPIs of PKAc with the phosphotransferase inhibiting RIIb and RIa subunits and the protein kinase inhibitor peptide (PKI). We analyzed PKA dynamics following integration of patient mutations into PKAc and RIa. We observed that oncogenic modifications of PKAc(L206R) and RIa(Δ184-236) as well as rare disease mutations in RIa(R368X) affect complex formation of PKA and its responsiveness to cAMP elevation. With the cell-based PKA PPI reporter platform we precisely quantified the mechanistic details how inhibitory PKA interactions and defined patient mutations contribute to PKA functions.

## Introduction

Small molecules represent the vast majority of cellular components. They are either substrates or products of numerous biochemical reactions. By acting as hormones or ligands, small molecules control protein functions, and molecular interactions which affect signal transmission. Molecular interactions of second messenger molecules with distinct proteins alter protein:protein interactions (PPIs) and signal propagation (Clapham, 2007; Scott and Pawson, 2009; Taylor et al., 2012; Li et al., 2013). The second messenger concept explains the dynamic wiring of membrane receptor pathways to a vast array of intracellular PPIs. One prominent example is the canonical second messenger molecule cyclic AMP (cAMP). It is an evolutionary conserved transmitter of membrane receptor originating input signals. A large number of G protein-coupled receptor (GPCR) cascade utilizes cAMP as intracellular second messenger (Neves et al., 2002; Pierce et al., 2002; Lefkowitz, 2007; O'Hayre et al., 2014). cAMP molecules dynamically bind to and regulate activities of its key effector kinase, the cAMP-dependent protein kinase A (PKA). The mechanistic details of cAMP-PKA activation is a textbook paradigm for allostery and small molecule:protein interactions (Taylor et al., 2012; Zhang et al., 2012). In its inactive state the PKA holoenzyme consists of a PKA regulatory subunit homodimer (R; type Ia, Ib, IIa, IIb) which inhibits the phosphotransferase activity of two PKA catalytic subunits (PKAc) through binding. Upon selective GPCR activation cAMP production through adenylyl cyclase (AC) activities is initiated; on the contrary, phosphodiesterase (PDE) control cAMP degradation (Dessauer, 2009; Houslay, 2010). cAMP binding to spatially compartmentalized type I (RI_2_:PKAc_2_) or type II (RII_2_:PKAc_2_) PKA holoenzymes causes a conformational change, hence dissociation, and activation of phosphotransferase activities of PKAc. As a consequence PKAc phosphorylates compartmentalized substrates which affects enzyme activities, PPI, localization, or protein abundance (Scott and Pawson, 2009; Lignitto et al., 2011, 2013; Taylor et al., 2012; Bachmann et al., 2013; Scott et al., 2013; Filteau et al., 2015). To guarantee substrate specificity PKA R subunits bind A kinase-anchoring proteins (AKAPs) which organize the subcellular targeting of PKA activities (Wong and Scott, 2004; Skroblin et al., 2010; Scott et al., 2013). To desensitize PKA phosphotransferase activities nuclear PKAc subunits are specifically inhibited through binding to the protein kinase inhibitor peptide (PKI) (Walsh et al., 1971; Scott et al., 1985; Dalton and Dewey, 2006). Deregulation of cAMP/PKA functions contribute to different diseases. Inactivating *PRKAR1A* gene (RIa) and activating *PRKACA* gene (PKAc) mutations/fusions provoke deregulated PKAc activities. As examples, mutations in RIa and PKAc account for the generation of endocrine tumors, cortisol-producing adenomas, and chimeric fusions with PKAc have been linked to the manifestation of hepatocellular carcinoma (Groussin et al., 2002; Meoli et al., 2008; Stratakis, 2013; Beuschlein et al., 2014; Calebiro et al., 2014; Cao et al., 2014; Di Dalmazi et al., 2014; Espiard et al., 2014; Goh et al., 2014; Honeyman et al., 2014; Sato et al., 2014; Cheung et al., 2015; Zilbermint and Stratakis, 2015). However, specific *PRKAR1A* gene (RIa) mutations impair cAMP-dependent PKA activation and cause hormone resistance as observed in the rare disease Acrodysostosis (a form of skeletal dysplasia) (Linglart et al., 2011; Assié, 2012; Silve et al., 2012). Here we tested the impact of functionally different PKA mutations on mutually exclusive molecular protein interactions using an extended PPI reporter platform based on the *Renilla* luciferase protein-fragment complementation assay (*R*luc PCA) (Stefan et al., 2007; Röck et al., 2015). We show that the presented *R*luc PCA platform—which is based on binary PKA network interactions—can be applied to analyze the consequences of patient mutations and upstream receptor activities on defined PKA PPIs directly in living cells.

## Materials and methods

### Cell culture and antibodies

HEK293 and U2OS cells were grown in DMEM supplemented with 10% FBS. Transient transfections were performed with Transfectin reagent (Biorad). Cells were treated with Forskolin (Biaffin) or Isoproterenol (Sigma) with indicated concentrations and for the indicated time frames. As primary antibody we used mouse anti-PKAc (BD Bioscience, #610981).

### Constructs

The *R*luc PCA based hybrid proteins RIIb-F[1] and PKAc-F[2] have been designed as previously described (Stefan et al., 2007). *R*luc PCA fusions with PKI and RIa have been generated using an analogous cloning approach. Following PCR amplification of the of the human RIa (alpha) gene (protein accession number: NP_002725.1) and PKI alpha gene (protein accession number: AAA72716; addgene plasmid # 45066) (Day et al., 1989) we fused them C-terminally with either -F[1] or -F[2] of the *R*luc PCA. PKA subunits and PKI were subcloned into the 5′ end the 10aa linker (GGGGS)_2_ and the *R*luc PCA fragments (pcDNA3.1 backbone vector). Site directed mutagenesis have been performed to generate the PKAc amino acid (aa) mutations G187V and L206R in the *PRKACA* gene and the RIa aa mutations R368X (X stands for the stop codon of the patient mutation; we generated the corresponding RIa truncation) in the *PRKAR1A* gene. In addition we deleted the corresponding nucleotide sequence from the aa 184–236 in the *PRKAR1A* gene.

### *Renilla* luciferase PCA of detached cells

HEK293 cells were grown in DMEM supplemented with 10% FBS. We transiently overexpressed indicated versions of the *R*luc PCA based reporter constructs in 24 or 12 well-plate formats. In case of the HEK293 cells we exchanged growth medium and resuspended cells in PBS 24 or 48 h post-transfection. Cell suspensions were transferred to 96-well plates and subjected to luminescence analysis using the LMax™-II-384 luminometer (Molecular Devices). *R*luc luminescence signals were integrated for 10 s.

### *Renilla* luciferase PCA of attached cells

We seeded U2OS cells into white-walled 96 well-plates with transparent bottom. We transiently overexpressed indicated versions of the *R*luc PCA based reporter constructs. 24 or 48 h post-transfection we exchanged growth medium with PBS. We performed luminescence analyses of attached U2OS cells at room temperature using the LMax™-II-384 luminometer (Molecular Devices). Time dependent changes of the *R*luc luminescence signals were integrated in 3-s intervals following addition of the *R*luc substrate benzyl-coelenterazine (5 μM; Nanolight) with or without Isoproterenol (final concentration 10 μM) (Stefan et al., 2007). Following determination of the *R*luc PCA signals we normalized the resulting signals on the control experiment (data points without Isoproterenol treatment).

## Results

**Figure 1:**
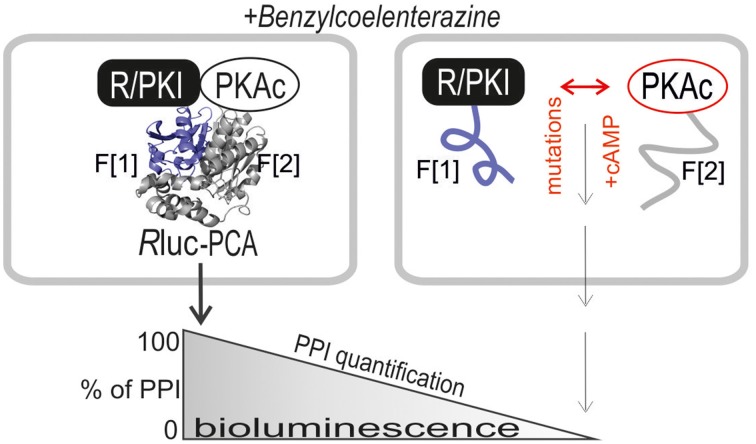
***R*luc PCA design principle**. The PKA subunits PKAc, RIa, and RIIb have been C-terminally tagged with *R*luc PCA fragments (-F[1], -F[2]). PPIs trigger folding and complementation of appended PCA-fragments. The emitted bioluminescence signal is a quantitative description of cellular PPIs. Elevations of cAMP levels and integration of distinct patient mutations in RIa and PKAc affect protein complex formation. Following addition of the *R*luc substrate benzyl-coelenterazine *R*luc PCA emitted bioluminescence signals reflect quantifiable PPI.

**Figure 2:**
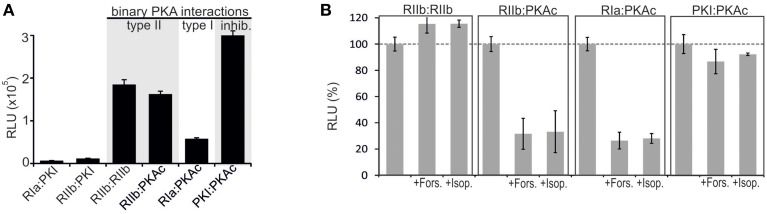
**Impact of receptor activities and kinase mutations on PPIs of PKA in human cell lines. (A)** Co-transfection of HEK293 cells with indicated *R*luc PCA pairs followed by *R*luc PCA analyses have been performed. The amount of PKI hybrid constructs have been bisected for cell transfections (representative of *n* = 5; ±SD from triplicates). **(B)** Indicated combinations of *R*luc PCA tagged components of the binary PKA network have been subjected to *R*luc PCA measurements. The effect of Forskolin (50 μM; 15 min) and Isoproterenol (10 μM; 15 min) on complex formation has been determined (average from at least *n* = 3 independent experiments; ±SEM). Basal PPIs of RIIb:RIIb, RIIb:PKAc, RIa:PKAc, and PKI:PKAc have been set to 100%. The percentage change of PPI is shown.

The purpose of our study was to extend the application spectrum of a PPI reporter platform (*R*luc PCA) to quantify mutually exclusive PKA interactions directly in living cells. It is the simplicity and sensitivity of the genetically encoded *R*luc PCA which enables different kinds of perturbation studies to unveil mechanistic details of PKA dynamics. The *R*luc PCA facilitates the precise quantification of cellular PPI in an appropriate *in vivo* setting (Röck et al., 2015). In Figure 1 we illustrate the *R*luc PCA design principle to analyze binary PPI of PKAc subunits. PPIs of R subunits and PKI with PKAc trigger folding and complementation of appended PCA-fragments. Following addition of the *R*luc substrate benzyl-coelenterazine *R*luc PCA emitted bioluminescence signals reflect quantifiable cellular PPI. Elevations of cAMP levels and defined patient mutations in RIa or PKAc affect PPI between the kinase inhibitory proteins (R/PKI) and PKAc which is indicated through alterations of bioluminescence signals. For the cloning of the PPI reporter constructs we fused the carboxy terminus of the PKA subunits (PKAc, RIIb, RIa) and PKI to complementary fragments of the *R*luc PCA (either -F[1] or -F[2]). In all cases the bait/prey proteins and the *R*luc PCA fragments are separated by an interjacent 10 amino acid (aa) linker with the sequence (GGGGS)_2_. It is a standard linker we previously showed to function using the dynamic RIIb:PKAc *R*luc PCA reporter (Stefan et al., 2007). We started with analyzes of time and dose dependent effects of distinct cAMP elevating agents on binary PKA interactions before we initiated investigations of the correlation of cAMP elevation, PPI and patient mutations. In our first tests we transiently over-expressed indicated *R*luc PCA pairs in HEK293 cells. We detected significant *R*luc PCA originating bioluminescence signals of existing PPI between RIIb:RIIb, RIIb:PKAc, RIa:PKAc, and PKI:PKAc. No significant bioluminescence signals with R subunit:PKI combinations were detectable (Figure 2A). These data confirm the specificity of the assay and the suitability to analyze PKAc complexes with differentially localized phosphotransferase inhibitors. PKA R subunits sense and bind cAMP. We tested different modes of cAMP elevation. We used the general cAMP elevating agent Forskolin to maximally activate AC mediated cAMP production. In addition, we activated a prototypical GPCR which is coupled to cAMP production, the beta-2 adrenergic receptor (β_2_AR), respectively. In this context it needs to be considered that distinct mechanism have been described how β_2_AR switch their G protein coupling from stimulatory G proteins to inhibitory ones (Baillie et al., 2003). We used HEK293 cells stably expressing β_2_AR. Activation of different βAR subtypes are related to proliferation, cardiac function, and memory and learning (Daaka et al., 1997; Wong and Scott, 2004; Zhang et al., 2005; Thaker et al., 2006; Lefkowitz, 2007). As predicted, we observed that both Forskolin exposure and the activation of β_2_AR (with Isoproterenol) triggered R:PKAc complex dissociation. We observed no major changes of PPI between PKI:PKAc and RIIb homodimers following 15 min of cAMP elevation (Figure 2B). These results indicate that discrete GPCR/cAMP activation induces dissociation of heterodimeric RIa:PKAc and RIIb:PKAc complexes which underlines the suitability of *R*luc PCA to characterize transient and small molecule regulated PPI *in vivo*.

**Figure 3:**
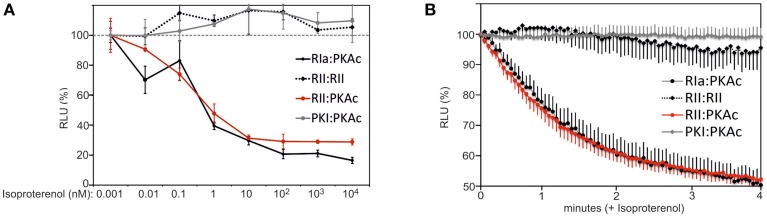
**Time and dose dependent impact of β_2_AR activities on PPIs. (A)** HEK293-β_2_AR cells transiently expressing indicated *R*luc PCA pairs have been exposed to dose-dependent Isoproterenol treatments (15 min). PPIs have been determined using the *R*luc PCA as read out (representative experiment from *n* = 3; ±SD from triplicates). PPIs of RIIb:RIIb, RIIb:PKAc, RIa:PKAc, and PKI:PKAc upon 1 pM Isoproterenol exposure have been set to 100%. **(B)** U2OS cells transiently expressing indicated *R*luc PCA pairs have been exposed to time-dependent Isoproterenol treatments (10 μM). Time dependent changes of the *R*luc luminescence signals were integrated for 3 s intervals following addition of the *R*luc substrate benzyl-coelenterazine (5 μM) with or without Isoproterenol. Following determination of *R*luc PCA signals we normalized the data points on the experiments with the full length *R*luc (±Isoproterenol) and on the control experiment (without Isoproterenol). Shown is a representative experiment of *n* = 3; ±SEM.

In order to evaluate dose dependent changes of type I and type II PKA dynamics we treated HEK293-β_2_AR cells with increasing concentrations of the non-selective beta adrenergic agonist Isoproterenol. We observed that similar half maximal effective concentrations of Isoproterenol induce PKA type I and type II activation in the used stable HEK293 cell line (Figure 3A). As predicted, we observed no major impact of β_2_AR-mediated cAMP elevation on RIIb homodimers and the PKI:PKAc complex. Next, we wanted to track time dependent changes of the studied PPIs. We switched to the well-attached human osteosarcoma cell line U2OS. We set out to monitor GPCR mediated changes of PPI in the first 4 min of agonist exposure of attached U2OS cells expressing endogenous β_2_AR directly in the 96-multiwell plate (Stefan et al., 2007). Following transient overexpression of the four *R*luc PCA pairs (RIa:PKAc, RIIb:PKAc, RIIb:RIIb, PKI:PKAc) and full length *R*luc we observed that Isoproterenol immediately induced the dissociation of heterodimeric type I and type II PKA complexes with similar kinetics. Again, β_2_AR activation for 4 min has no major effect on the RIIb dimer and on PKAc:PKI interactions (Figure 3B). The results from the real-time measurements of PKA kinetics using whole cell populations underline the suitability of the *R*luc PCA to compare subtype-receptor controlled PPI dynamics in distinct cell settings.

**Figure 4:**
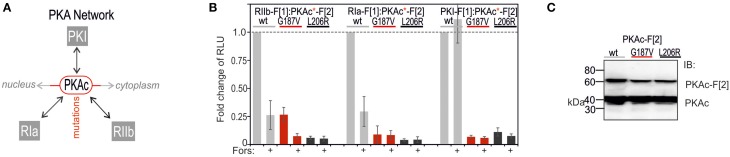
**Impact of PKAc mutations and cAMP elevation on PPIs emanating from PKAc. (A)** cAMP elevation leads to PKA activation: cAMP binds to R subunits and triggers dissociation of active PKAc subunits which phosphorylate substrates in cytoplasm and nucleus. PKI inactivates nuclear PKI complexes. PKAc mutations deregulate the binary PKA network. **(B)** Following site directed mutagenesis of PKAc-F[2], combinations of wild type and mutant *R*luc PCA tagged PKA subunits have been subjected to *R*luc PCA measurements. The effect of forskolin (50 μM; 10 min) on PKA complex formation has been determined (*n* = 4 independent experiments, ±SEM). **(C)** Immunoblotting shows expression levels of endogenous PKAc and F[2]-tagged PKAc variants.

Next, we analyzed different kind of patient mutations of PKA subunits and their impact on binary PKA network interactions. We selected patient mutations of PKAc and RIa which contribute to disease etiology and progression (Carney et al., 1985; Horvath et al., 2010; Stratakis, 2013; Beuschlein et al., 2014; Calebiro et al., 2014; Cao et al., 2014; Di Dalmazi et al., 2014; Espiard et al., 2014; Goh et al., 2014; Honeyman et al., 2014; Salpea and Stratakis, 2014; Sato et al., 2014; Cheung et al., 2015; Zilbermint and Stratakis, 2015). We started with analyses of PKAc mutations and their effect on compartmentalized PPI of PKAc with R subunits (type I and II) and PKI (Figure 4A). In addition we investigated the impact of cAMP alterations on wild type and mutant PKA complexes. We introduced two patient mutations into the PKAc-F[2] *R*luc PCA constructs. It has been described that the PKAc mutation G187V diminished, whereas the hotspot mutation L206R of PKAc in adrenal Cushing's syndrome promoted PKAc activity (Soberg et al., 2012; Cao et al., 2014; Di Dalmazi et al., 2014; Sato et al., 2014). Following co-expression of PCA pairs in HEK293 cells we observed a significant reduction of complex formation of PKAc mutants with all three PKA-inhibitory proteins. To our surprise also the interaction with PKI was significantly reduced. cAMP binding to the heterodimeric wild type PKA reporter (RIa:PKAc, RIIb:PKAc) caused complex dissociation (Stefan et al., 2007, 2011; Bachmann et al., 2013). Only in the case of RIIb:PKAc-G187V the remaining PPI was still responsive to general cAMP elevation using Forskolin (Figure 4B). The expression level of mutated PKAc-F[2] was slightly reduced (Figure 4C). These data underline that the patient mutation PKAc-L206R and the mutant PKAc-G187V significantly reduce complex formation with RIa, RIIb, and PKI. However, we show that RIIb has still the potential to bind to the catalytic inactive PKAc-G187V mutant. No remaining PPIs of inhibitory R/PKI proteins with the critical PKAc-L206R mutant were detected.

**Figure 5:**
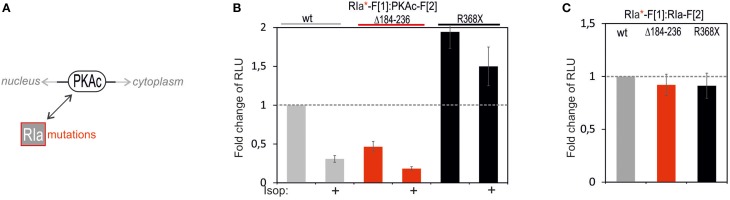
**Impact of RIa mutations and cAMP elevation on PKA type I complexes. (A)** Ligand-mediated activation of GPCR pathways linked to cAMP production lead to activation of PKA holoenzymes. cAMP binds to RIa subunits and triggers dissociation of active PKAc subunits which phosphorylate substrates in cytoplasm and nucleus. RIa mutations deregulate RIa:PKAc dynamics. **(B)** Following indicated modifications of RIa-F[1] sequences, combinations of wild type and mutant *R*luc PCA tagged PKA subunits have been subjected to *R*luc PCA measurements. The effect of isoproterenol (1 μM, 10 min; 48 h transient PCA reporter expression) on PKA complex formation has been determined (at least *n* = 3 independent experiments, ±SEM). **(C)** Analyses of *R*luc PCA based homodimer formations of wild type and mutated RIa have been performed (at least *n* = 3 independent experiments, ±SEM).

Following analyses of PKAc patient mutations we tested the consequence of mutating RIa on complex formation with PKAc (Figure 5A). In the RIa R subunit a variety of mutations have been found which lead to distinct human diseases (Figure 5A) (Horvath et al., 2010). We decided to test a patient mutation which causes the expression of a RIa protein lacking the aa sequence encoded by exon 6 (R1a Δ184-236) which contribute to the multiple neoplasia and lentiginosis syndrome Carney complex (Groussin et al., 2002; Meoli et al., 2008). It is a disease described as “the complex of myxomas, spotty skin pigmentation and endocrine over activity” (Carney et al., 1985; Veugelers et al., 2004; Horvath et al., 2010; Salpea and Stratakis, 2014). In case of the Carney complex a collection of RIa mutations lead to PKA activation. As second mutation we introduced one stop codon at the position R368X in RIa which results in a truncation of the last 14 amino acids of the RIa protein. This heterozygous *de novo* mutation has been identified in patients with Acrodysostosis (a rare form of skeletal dysplasia) which is caused through a resistance to several hormones (Linglart et al., 2011). In case of Acrodysostosis RIa mutations lead to PKA inactivation.

To quantitatively evaluate the impact of these two patient mutations on PPI we transfected the same DNA amount of indicated *R*luc PCA constructs into HEK293 cells. We observed that the R1a Δ184-236 deletion in RIa-F[1] reduced basal PPI with PKAc-F[2] to less than 50% (Figure 5B). No major changes of R subunit dimerization of R1a Δ184-236:RIa was detectable (Figure 5C). Upon cAMP elevation with Isoproterenol the remaining PKA complex can be activated to a similar extend as the wild type PKA type I complex. In parallel we analyzed the truncated RIa protein simulating the stop codon at position R368 of RIa (= 14aa truncation). First, we observed elevated levels of inactive RIa:PKAc complexes. Second, upon cAMP elevation with Isoproterenol we recorded less PKA dynamics (Figure 5B). No major changes of R subunit dimerization of RIa-R368X:RIa was detectable (Figure 5C). Apparently the RIa-R368X mutated PKA type I complex is less responsive to cAMP elevations. The R368X mutation impairs the cAMP-dependent dissociation of RIa from PKAc. These data underline that specific patient mutations in RIa have different consequences on both the PKA holoenzyme complex formation and on cAMP-induced PKA activation.

Overall our data indicate that the presented *R*luc PCA PKA reporter platform is suitable to quantify dynamic and mutually exclusive protein complex assembly and disassembly of the binary PKA network, which is controlled through oscillations of cAMP levels. In addition, we clearly demonstrate with this proof of concept study that observed PPI dynamics of wild type and mutated PKA complexes support findings and hypotheses of disease relevant PKA associations.

## Discussion

The ability to precisely record PPI dynamics of the binary PKA network is central for understanding compartmentalized cAMP signal transmission. We present a highly sensitive and easy applicable reporter platform to systematically quantify, map, and manipulate PKA protein complex formation in real time. We show that second messenger initiated changes of PKA reflect connections to upstream located receptor pathways. The implementation of the PPI reporter allows for more accurate and quantitative specification of receptor-effector relationships directly in the preferred cell type. In light of disease relevant PKAc variations (mutations, fusions, decontrolled upstream pathways) application of the PKA *R*luc PCA platform offers the possibility to systematically test different means of kinase perturbations using the available biosensor toolbox. We quantified mutually exclusive PKAc interactions which account for different spatially controlled cell functions. We showed that the PPI reporter implementation simplified the determination of consequences of PPI relevant patient mutations. Such comparative *in vivo* analyses will facilitate the decision how to interfere with deregulated PKA functions. We showed that two PKAc mutations display differences in the binary PPI pattern. We revealed that cAMP-sensing R subunits and PKI form no PPIs with PKAc-L206R, a hotspot mutation of PKAc in adrenal Cushing's syndrome which promotes PKAc activity (Cao et al., 2014; Di Dalmazi et al., 2014). First, this implements that PKAc-L206R cannot be compartmentalized through R/PKI and indirect AKAP interactions. Second, PKAc-L206R operates independently of cAMP. Therefore, alternative means need to be prosecuted to reduce the deregulated phosphotransferase activity. Besides inhibitory small molecules we believe that targeting the stability of PKAc-L206R is one alternative.

We also tested two patient mutations of RIa which are related to two different diseases showing opposite effects on PKA activity. We demonstrated that the Carney complex relevant R1a-Δ184-236 mutation had a major impact on PKA complex formation when compared to the wild type PKA holoenzyme type I. Our data showed that R1a-Δ184-236 has less affinity for PKAc. We assume that the reduced affinity for PKAc contributes to disease relevant elevations of PKAc phosphotranferase activities. The second mutation RIa-R368X (= 14aa truncation) caused a defect in PKA activation. The mutation introduces a stop codon and shortens the cAMP binding domain B. The resulting deletion of 14 aa at the C terminus reduced the cooperative binding of cAMP first to binding domain B and then to binding domain A, which is a prerequisite for PKAc dissociation and activation (Kim et al., 2007; Bertherat et al., 2009; Linglart et al., 2011; Taylor et al., 2012). We showed that this truncation is sufficient to elevate basal RIa-1-368:PKAc complexes. Primarily this complex was less responsive to GPCR/cAMP mediated PKA activation what correlates with the findings of Linglart and colleagues (Linglart et al., 2011; Silve et al., 2012). Interestingly, we detected marginal cAMP-dependent activation of the mutated PKA complex. This underscores the suitability of the PKA *R*luc PCA reporter to detect even modest changes of PPIs in response to second messenger elevations. Overall we underline with this study that the *R*luc PCA is a versatile reporter system to map transient and small molecule controlled PPI. Besides linking membrane receptor pathways to alterations of molecular interactions, the consequence of patient mutations on PPI can be tested with a simple protocol and in parallel fashion. The implementation of this PPI reporter platform will unveil consequences of distinct patient mutations on binary PKA network dynamics. We envision that the mutation approach will disclose hot spots in these critically regulated kinase complexes which become pharmaceutical targets to target both PPI and/or cAMP sensing. This will help to gain novel insights into mechanism of PKA activation which will be relevant for the diagnosis and for identifying treatments of kinase involved diseases.

## Author contributions

RR, JM, VB, and ES performed the experiments. RR, JM, VB, and ES analyzed the results. ES wrote the manuscript.

### Conflict of interest statement

The authors declare that the research was conducted in the absence of any commercial or financial relationships that could be construed as a potential conflict of interest.
