# Corrigendum: Impact of kinase activating and inactivating patient mutations on binary PKA interactions

**DOI:** 10.3389/fphar.2015.00214

**Published:** 2015-09-24

**Authors:** Ruth Röck, Johanna E. Mayrhofer, Verena Bachmann, Eduard Stefan

**Affiliations:** Institute of Biochemistry and Center for Molecular Biosciences, University of InnsbruckInnsbruck, Austria

**Keywords:** molecular interactions, patient mutations, cAMP-dependent protein kinase A, protein-fragment complementation assay, GPCR, Carney complex, Acrodysostosis

Reason for Corrigendum:

There was a mistake in the figure legend for **Figure 5** as published. The correct version of the figure legend **(Figure 5B)** appears below. The authors apologize for the mistake. This error does not change the scientific conclusions of the article in any way.

**Figure 5. Impact of RIa mutations and cAMP elevation on PKA type I complexes**. … **(B)** Following indicated modifications of RIa-F[1] sequences, combinations of wild type and mutant *R*luc PCA tagged PKA subunits have been subjected to *R*luc PCA measurements. The effect of isoproterenol (1 μM, 10 min; 48 h transient PCA reporter expression) on PKA complex formation has been determined (at least *n* = 3 independent experiments, ±SEM).

The original article was updated.

## Conflict of interest statement

The authors declare that the research was conducted in the absence of any commercial or financial relationships that could be construed as a potential conflict of interest.

